# Challenges and opportunities to minimize the dose in the neurovascular bundles during prostate radiotherapy

**DOI:** 10.1016/j.ctro.2025.100959

**Published:** 2025-04-09

**Authors:** Victor J. Brand, Linda Rossi, Maaike T.W. Milder, Femke E. Froklage, Alison C. Tree, Mischa S. Hoogeman, Luca Incrocci

**Affiliations:** a Erasmus MC Cancer Institute, University Medical Centre Rotterdam, Department of Radiotherapy, UK; b The Royal Marsden Hospital, London, UK; c The Institute of Cancer Research, London, UK

**Keywords:** Prostate cancer, Neurovascular bundle, SBRT, Erectile dysfunction, Treatment planning

## Abstract

Background and purpose: Radiation damage to the neurovascular bundles (NVB) has been linked to erectile dysfunction after prostate cancer radiotherapy (PCa). NVB sparing using coplanar and non-coplanar automated treatment planning is presented here in two settings: (1) without compromising target coverage, (2) allowing target coverage compromise. Material and methods: 20 previously treated patients with localized PCa. Based on a MRI-CT match, the NVB were retrospectively delineated. All treatment plans (5 × 7.25 Gy) were automatically generated using Erasmus-iCycle (in-house automated treatment planning algorithm). Non-NVB sparing (non-NVBsparing) plans and NVB sparing plans in two settings were generated: (1) uncompromised NVB sparing (u-NVBsparing; maintaining target coverage) (2) and compromised NVB sparing (c-NVBsparing; allowing target underdosage). Coplanar and non-coplanar beam arrangements were compared. U-NVBsparing was compared to non-NVBsparing. C-NVBsparing plans were visualized in Pareto fronts. Statistical significance (p-value < 0.05) was determined by Wilcoxon signed-rank test. Results: u-NVBsparing compared to non-NVBsparing plans showed statistically significant median reductions in NVB D0.1 cc (38.9 vs 42.6 Gy for coplanar; 38.9 vs 43.3 Gy for non-coplanar) and Dmean (25.6 vs 30.0 Gy for coplanar; 24.7 vs 30.2 Gy for noncoplanar). Further lowering NVB D0.1 cc in c-NVBsparing plans clearly correlated to lower target coverage. Non-coplanar c-NVBsparing plans maintained significantly higher target coverages for similar NVB D0.1 cc values, compared to coplanar plans. Conclusion: NVB sparing without compromising target coverage is feasible. No clinically relevant benefit was found for non-coplanar compared to coplanar NVB sparing plans, although overall statistically superior. Further sparing of the NVB comes at the cost of target coverage, for which a Pareto front could be used as a tool in clinical practise.

## Highlights

• NVB sparing without compromising target coverage is feasible.
• Non-coplanar compared to coplanar NVB sparing plans showed lower OAR doses, especially in more challenging treatment plans.
• Eventually NVB sparing comes at the cost of target coverage, which can be visualized by a Pareto front.
• Pareto frons can be used as a tool for shared decision making in clinical practice.

## Introduction

Radiotherapy is one of the main treatment modalities to treat prostate cancer [1] and has shown an ongoing shift towards (ultra-) hypofractionation, due to the relatively low alpha/beta ratio of prostate cancer [2], [3]. However, higher doses per fraction require advanced treatment planning and image-guidance to avoid high doses in normal tissues and reduce possible negative side-effects. One major side effect is erectile dysfunction (ED), occurring in approximately 40–55 % of irradiated patients [2], [3], which can have a significant impact on quality of life after treatment [4]. The mechanism for ED after radiation therapy remains unclear, and multiple penile structures have been suggested to play a role. Sparing penile structures, such as penile bulb, crus [5], [6], and internal pudendal arteries [5], [6], [7], have shown mixed results. However, neurovascular bundle (NVB) sparing has been suggested to play a role in preservation of erectile function after radiation therapy [8], [9] and prostatectomy [10], [11] both. But, much is still unknown regarding feasibility of sparing the NVB while using ultra-hypofractionation and the extend of dose reduction needed for clinical benefit.

Previously, sparing the NVB was thought to be unrealistic, considering their position directly abutting the prostate [12]. However, with recent technical advancements in treatment planning and image-guidance [13], NVB sparing is possible. The use of non-coplanar beam angles in particular has shown dosimetric advantages for sparing the pelvic organs at risk (OARs) during prostate ultra-hypofractionation [14], [15], which could translate into a NVB sparing setting as well.

Automated treatment planning is a powerful tool to investigate possible protocol modification or improvements. It allows to objectively investigate trade-offs between clinical criteria and compare different treatment strategies (e.g. beam arrangement) without planner experience and available time bias [16], [17].

A recent planning study has shown feasibility of NVB sparing using MR-guided radiotherapy, resulting in significantly lower NVB D_0.1cc_ [18]. In the plans, which had homogenous target dose distributions, the target coverage aims were altered to facilitate NVB sparing.

To our knowledge, this is the first study to investigate the feasibility of NVB sparing in prostate SBRT using automated treatment planning in two different settings: (1) without compromising target coverage and (2), in light of shared decision making, by investigating trade-offs between NVB dose and target coverage using Pareto fronts. During this process, plans based on coplanar and non-coplanar beam arrangements have been investigated.

## Material and methods

### Patient selection

Previously treated patients on the CyberKnife system, with a CT-scan (CT voxel size 0.98 x 0.98 x 1.5 mm^3^) for treatment planning and T2-weighted MRI sequences (1.5 T) for contouring, were eligible for inclusion in this retrospective study. While excluding patients with fiducial markers overlapping the posterolateral prostate border or patients with MRI-scans lacking sufficient quality for target- and organs at risk delineation, 20 patients were randomly selected. Written informed consent was previously obtained for all included patients.

*Image registration and delineation:* For all 20 patients, the T2-weighted MRI was rigidly registered, under supervision of an experienced radiation technician with the CT-scan using the intraprostatic urethra as reference. The NVB and the prostate were delineated on the planning MRI by the main investigator (VB) under guidance of a pelvic radiologist, allowing only minor changes of these MRI contours based on anatomical boundaries on the planning CT (e.g. NVB partially overlapping muscle). The NVB were defined in accordance with the POTEN-C trial (clinicaltrials.gov; NCT03525262): two separate structures as visualized on the MRI, including their extension up to 1 cm cranially and caudally from the PTV. The remaining OARs (bladder, rectum and bowel) were delineated on the planning CT-scan. See Table A1 (Appendix A) for full list of delineation definitions.

### Treatment plan generation and clinical protocol

Simulated treatments consisted of 5 fractions of 7.25 Gy, with 36.25 Gy prescribed to 95 % of the planning target volume (PTV, prostate + 3 mm), and 40 Gy prescribed to 95 % of the prostate volume. Treatment plans were automatically generated using Erasmus-iCycle [19], an in-house developed automated treatment planning system in which a wish-list of constraints and objectives is compiled to adhere to the clinical protocol. A reference wish-list was created to automatically generate plans fulfilling the dosimetric planning aims of the PACE trial [20], [21] (see Table B1, appendix B for the full list of planning constraints and objectives). After target V_95%_ coverage for both PTV and prostate, rectum and bladder sparing at high doses were the first priorities, followed by mean dose of urethra, bladder, rectum and near high dose of the femur.

Coplanar and non-coplanar plans were automatically generated based on two previously validated class solutions. For coplanar plans, 23 equi-angular beams in the axial plane through the isocentre, covering 360° around the patient was used, simulating volumetric arc therapy (VMAT) treatments [22]. For non-coplanar plans, a 25 non-coplanar beam class-solution for SBRT prostate cancer was developed obtaining comparable quality to patient-specific optimally selected non-coplanar beam angles [23].

### Generated plans

First, a reference wish-list was generated to create plans fulfilling the standard clinical protocol, i.e. without sparing of NVB. Those reference plans were referred to as ‘*non-NVBsparing*’ plans (reference plans). Coplanar and non-coplanar non-NVBsparing plans were automatically generated with the reference wish-list reported in Table B2 (appendix B). The reference wish-list was equal for all patients and without patient-specific tuning or modification. Subsequently, two sets of NVB sparing plans were generated: (1) aiming to achieve NVB sparing while maintaining target coverage (uncompromised sparing plans: *‘u-NVBsparing’*) and (2) aiming for further NVB sparing while accepting possible target coverage loss (compromised sparing plans: ‘*c-NVBsparing’*). Automated u-NVBsparing plans were generated by adding an equivalent uniform dose objective for both NVBs to the wish-list, i.e. aiming to reduce high dose in the NVBs, after achieving PTV and prostate coverages. Automated c-NVBsparing plans were generated by turning the NVB objective into a NVB D_max_ constraint, i.e. NVB doses always achieved at possible cost of lower target coverages. As the NVB consist of neural and vascular tissues, NVB sparing mainly focused on reducing the near max dose (D_0.1cc_) [24]. To visualize the compromise between NVB sparing and target coverage, the NVB D_max_ constraint was lowered stepwise from 50 Gy (equal to non-NVBsparing situation) to 25 Gy using intermediate values of 40, 37.5, 35, 32.5, and 30 Gy to create a 2D Pareto front. This process was repeated for coplanar and non-coplanar treatment plans.

### Plan comparison and statistical analyses

All plans were rescaled to PTV V_36.25Gy_ = 95 %, if ≤95 %, for plan acceptability (with exception to c-NVBsparing plans where underdosage was desired). As per PACE trial protocol [20], [21], prostate V_40Gy_ and PTV V_36.25Gy_ were collected for target coverage and urethra, rectum, bladder, bowel, femoral and, for this study specifically, NVB dosimetric parameters were collected for the OARs, as reported in Table B1. Non-NVBsparing, u-NVBsparing and c-NVBsparing plans were compared on differences between coplanar and non-coplanar beam arrangements. Furthermore, the u-NVBsparing plans were compared to the non-NVBsparing plans. The c-NVBsparing plans, possible trade-offs between NVB D0.1 cc and PTV coverage, prostate coverage and OAR doses were examined as well. A Wilcoxon signed rank test was used for plan comparison in R (version 4.2.1), using a statistical significance level of a p-value < 0.05.

## Results

### Non NVB sparing plans (reference plans)

Table 1 shows an overview of the extracted dosimetric parameters of the non-NVBsparing plans. All plans adhered to target and OAR PACE constraints, except for one non-coplanar plan, which exceeded the urethra dose (V_42Gy_ of 51.5 % instead of < 50 %, notably this patient had the smallest urethra volume).

**Table 1:** Planned dose comparison on relevant dosimetric parameters between non NVB sparing (non-NVBsparing) and uncompromised NVB sparing (u-NVBsparing) plans for both coplanar and non-coplanar beam arrangements. Statistical significance between non NVB sparing and uncompromised NVB sparing plans was determined using Wilcoxon signed rank test. IQR = interquartile range; PTV = planning target volume; NVB = Neurovascular Bundles (left and right were combined); Gy = Gray.

			**Coplanar beam arrangement**			**Non-coplanar beam arrangement**		
**Structure**	**Parameter**		**non-NVB sparing**	**u-NVB sparing**		**non-NVB sparing**	**u-NVB sparing**	
	(PACE-constraint^23^)	Unit	Median (IQR)	Median (IQR)	P-value	Median (IQR)	Median (IQR)	P-value
**PTV**	**V_36.25Gy_** (>95 %)	%	97.3 (97.0–97.7)	95.6 (95.2–95.8)	<0.001	97.2 (97.0–97.6)	95.4 (95.1–95.7)	<0.001
**Prostate**	**V_40Gy_** (>95 %)	%	97.7 (97.4–98.2)	95.9 (95.8–96.2)	<0.001	97.7 (97.4–98.1)	95.8 (95.8–96.2)	<0.001
**NVB**	**D_0.1cc_**	Gy	42.6 (42.4–43.2)	38.9 (38.3–39.1)	<0.001	43.3 (43.1–43.5)	38.9 (38.3–39.1)	<0.001
	**D_mean_**	Gy	30.0 (28.9–31.6)	25.6 (23.4–26.2)	<0.001	30.2 (28.1–30.7)	24.7 (22.5–26.0)	<0.001
**Urethra**	**V_42Gy_** (<50 %)	%	40.1 (36.6–44.3)	40.8 (38.4–46.4)	0.053	41.8 (38.5–43.4)	41.6 (39.5–44.3)	0.56
**Rectum**	**V_36Gy_** (<2cc)	cc	0.23 (0.07–0.44)	0.45 (0.24–0.66)	<0.001	0.20 (0.06–0.38)	0.41 (0.23–0.65)	<0.001
	**V_29Gy_** (<20 %)	%	3.2 (2.4–5.1)	3.9 (2.8–5.6)	<0.001	3.0 (2.3–4.8)	3.6 (2.6–5.3)	<0.001
	**V_18.1Gy_** (<50 %)	%	10.3 (6.8–15.0)	12.8 (8.3–16.3)	<0.001	9.4 (6.2–13.4)	10.6 (7.1–14.3)	<0.001
**Bladder**	**V_37Gy_** (<10 cc)	cc	2.90 (1.91–3.78)	3.28 (2.45–4.41)	<0.001	3.00 (1.92–3.98)	3.36 (2.45–4.51)	<0.001
	**V_18.1Gy_** (<40 %)	%	13.7 (12.1–17.8)	14.9 (12.8–19.9)	<0.001	11.6 (10.6–13.1)	12.8 (11.6–14.6)	<0.001
**Bowel**	**V_30Gy_** (<1cc)	cc	0.0 (0.0–0.0)	0.0 (0.0–0.0)	1	0.0 (0.0–0.0)	0.0 (0.0–0.0)	1
	**V_18.1Gy_** (<5cc)	cc	0.0 (0.0–0.0)	0.0 (0.0–0.0)	0.37	0.0 (0.0–0.0)	0.0 (0.0–0.0)	1
**Femurs**	**V_14.5Gy_** (<5%)	%	2.3 (1.5–3.1)	2.0 (1.2–3.2)	0.11	0.2 (0.0–0.5)	0.0 (0.0–0.2)	0.009

### Uncompromised NVB sparing plans

Table 1 also shows an overview of the extracted dosimetric parameters of the u-NVBsparing plans. Prior to scaling, three patients showed a slight PTV V_36.25Gy_ underdosage in all u-NVBsparing plans (coplanar and non-coplanar) with the minimum being 94.7 %. After scaling, three patients showed OAR dose constraints violations. For two patients the urethra constraint (V_42Gy_ < 50 %) was violated, for one of those patients only in the coplanar sparing plan (V_42Gy_ was 50.9 %) and the second of those two patients for both non-coplanar and coplanar plans (V_42Gy_ of 51.7 % and 53.2 % respectively). In a third patient, the bladder V_37Gy_ < 10 cc constraint was exceeded in both coplanar and non-coplanar plans (V_37Gy_ of 10.8 and 11.2 cc respectively).

### Non NVB sparing vs. uncompromised NVB sparing plans

**Fig. 1:**
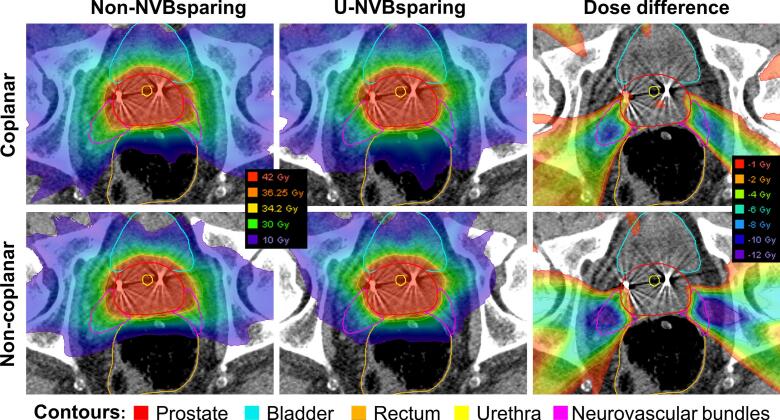
Example of a 2D dose distribution of the non NVB sparing (left) and scaled uncompromised NVB sparing (middle) and absolute dose difference between these two (right) for both coplanar (top) and non-coplanar (bottom) beam arrangements for one patient. Non-NVBsparing = Non NVB sparing plans. U-NVBsparing = uncompromised NVB sparing plans.

**Fig. 2:**
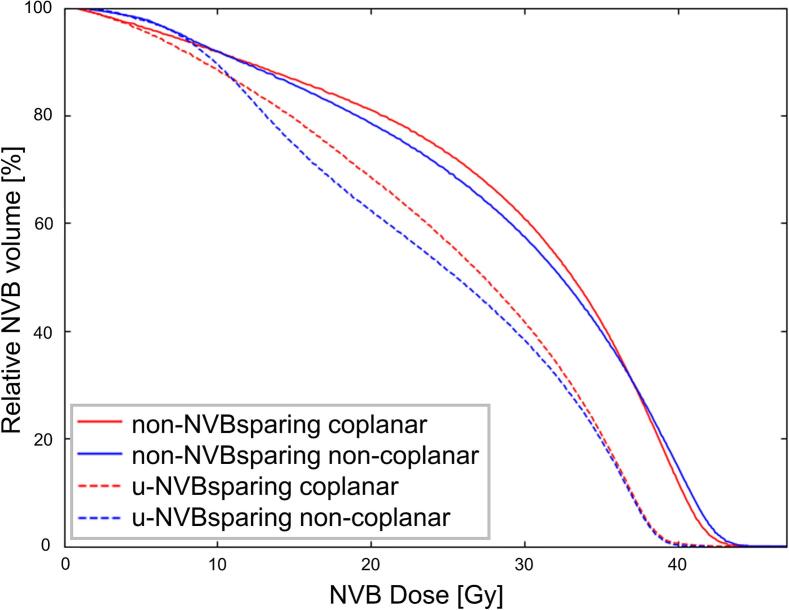
Population average dose volume histograms of both neurovascular bundles for coplanar and non-coplanar plans. NVB = neurovascular bundles; Gy = Gray. Non-NVBsparing = Non NVB sparing plans. U-NVBsparing = uncompromised NVB sparing plans.

Non-NVBsparing and u-NVBsparing plan comparison, both coplanar and non-coplanar beam arrangements, is reported as population median values in Table 1, as dose distribution comparison for an example patient in Fig. 1, and as NVB population dose volume histograms (DVHs) in Fig. 2. U-NVBsparing plans showed statistically significant reductions compared to non-NVBsparing plans in NVB D_0.1cc_ of median 38.9 vs 42.6 Gy, for coplanar plans, and median 38.9 vs 43.3 Gy, for non-coplanar plans. Similarly for NVB D_mean_ statistically significant reductions were seen comparing u-NVBsparing to non-NVBsparing, for both coplanar and non-coplanar (25.6 vs 30.0 Gy and 24.7 vs 30.2 Gy respectively) as shown in Table 1 and Fig. 2. In contrast, u-NVBsparing plans had less optimal prostate V_40Gy,_ PTV_V36.25Gy_ and rectum and bladder dose parameters compared to non-NVBsparing, but still well within PACE constraints (Table 1), similar for both coplanar and non-coplanar.

### Coplanar vs. non-coplanar (non-NVBsparing and u-NVBsparing)

When looking at non-NVBsparing plans (Table C1), the difference in PTV V_36.25Gy_ between coplanar and non-coplanar plans was statistically significant but clinically not relevant (median 97.3 % and 97.2 % respectively, p < 0.001). Prostate V_40Gy_ did not differ significantly between non-coplanar and coplanar plans (both 97.7 %, p = 0.13). Rectum (V_36Gy_, V_29Gy_ and V_18.1Gy_), femurs (V_14.5Gy_) and bladder V_18.1Gy_ were statistically significantly lower with non-coplanar plans compared to coplanar plans, with reduction up to 2.1 % in median values (all p-values < 0.001, see more details in Table C1). Only bladder V_37Gy_ was statistically significantly higher for non-coplanar compared to coplanar plans (median V_37Gy_ 3.0 and 2.9 cc respectively, p = 0.003).

For u-NVBsparing plans, the same conclusion holds when comparing coplanar and non-coplanar plans. With targets, urethra, bowel and femurs being similar between coplanar and non-coplanar plans, while rectum and bladder dose is lower in non-coplanar plans. In addition, non-coplanar plans did show a significantly lower NVB D_mean_ compared to coplanar plans (24.7 vs 25.6 Gy respectively, p < 0.001), while no significant difference was found for NVB D_0.1cc_ between coplanar and non-coplanar NVB sparing.

### Compromised NVB sparing plans (Pareto front)

**Fig. 3:**
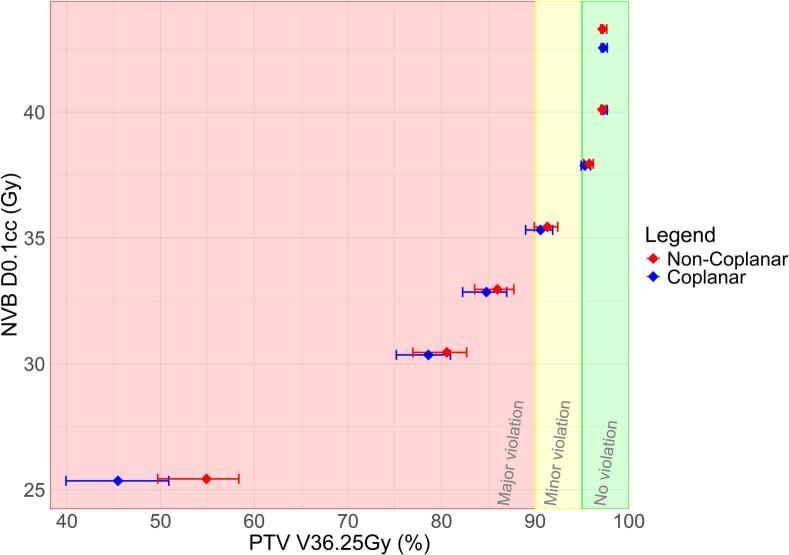
Point graph of compromised NVB sparing plans with interquartile ranges error bars, showing the trade-off between population median NVB D0.1 cc and PTV V36.25 Gy coverage for a stepwise reduction in the NVB Dmax constraint. Green area = adhering to target PTV coverage; yellow area = minor violation to target PTV coverage; Red area = major violation to target PTV coverage. NVB = Neurovascular bundles; PTV = Planning target volume. (For interpretation of the references to colour in this figure legend, the reader is referred to the web version of this article.)

**Fig. D1:**
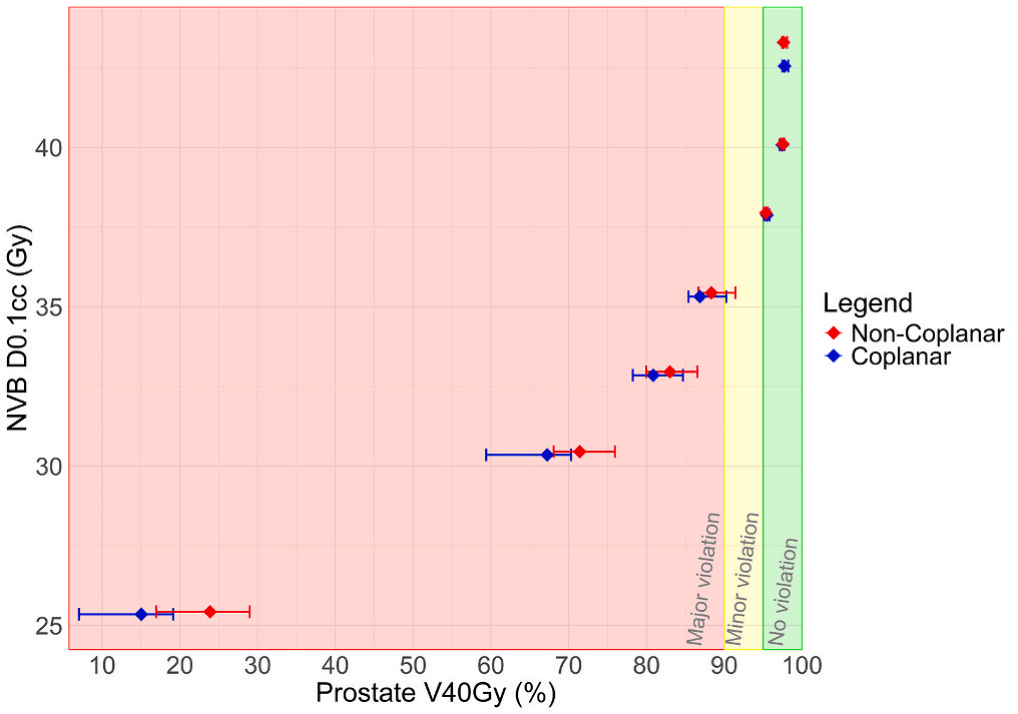
Point graph of compromised NVB sparing plans with interquartile ranges error bars, showing the trade-off between population median NVB D_0.1cc_ and prostate V_40Gy_ coverage for a stepwise reduction in the NVB D_max_ constraint. Green area = adhering to target PTV coverage; yellow area = minor violation to target PTV coverage; Red area = major violation to target PTV coverage. NVB = Neurovascular bundles; PTV = Planning target volume.

The trade-off between NVB D_0.1cc_ versus PTV coverage was visualized by a Pareto front (Fig. 3). The starting point in the Pareto front was formed by plans with a NVB constraint exceeding the maximum dose in the plans, hence without any sparing of the NVB (i.e. non-NVBsparing plans). By stepwise lowering this NVB constraint, a clear correlation was observed between NVB D_0.1cc_ and PTV coverage. The green area in Fig. 3 represents plans that adhered to the clinical PTV V_36.25Gy_ constraint of >95 % Gy. The yellow area in Fig. 3 represents plans with a minor violation (as defined in the PACE protocol [21]): PTV V36.25 Gy between 90 and 94.9 %. Multiple plans in these areas showed NVB D_0.1cc_ reductions with no or minor violations to the requested PTV coverage of V_36.25Gy_ > 95 %. A relationship similar to the PTV V_V36.25Gy_ was observed between prostate V_40Gy_ and NVB _D0.1cc_ (see Fig. D1). Multiple plans showed a decrease in NVB D_0.1cc_ without a significant reduction in prostate V_40Gy_ below 95 % (Fig. D1, green and yellow areas).

All OARs showed an initial increase in dose when stepwise reducing the NVB D_max_ constraint, due to higher complexity in multi-criteria problem where a strong NVB D_max_ constraint and still feasible target coverage coexist. In this phase, some OAR requirement violations were seen, indicating that rescaling of those plans would have been necessary if clinically applied. When lowering the NVB D_max_ constraint further, thereby compromising the target dose, a significant decrease in OAR dose was seen as well, the onset of which depended on the actual dose constraint of the OAR (more details are reported in Tables E1 and E2).

When looking at coplanar vs non-coplanar c-NVBsparing plans, non-coplanar plans showed higher PTV V_36.25Gy_ for all NVB constraint value plans, compared to coplanar plans. These differences increased in favour of non-coplanar plans, with decreasing NVB D_max_ constraint (median PTV V_36.25Gy_ of 45.4 % versus 54.7 % respectively when the NVB D_max_ constraint was set to 25 Gy, p < 0.001). Similarly, the differences between prostate V_40Gy_ in coplanar and non-coplanar were largest for the lowest value of the NVB D_max_ constraint of 25 Gy (median 12.7 vs 23.8 % respectively, p < 0.001), in favour of non-coplanar plans. The differences gradually disappeared towards the highest value of NVB D_max_ of 50 Gy (97.7 % vs 97.7 %, p = 0.13).

## Discussion

To our knowledge, this is the first study describing NVB sparing using automated planning for both non-coplanar and coplanar beam arrangements. NVB sparing without compromising clinical constraints is shown to be feasible, showing significant reductions in median NVB D_0.1cc_ of 3.7 and 4.4 Gy and median D_mean_ of 4.4 and 5.5 Gy for coplanar and non-coplanar, respectively. Although overall statistically superior, no clinically relevant improvement was found comparing non-coplanar to a coplanar beam arrangement for uncompromised NVB sparing, except for a clear and possible, albeit unproven, clinical benefit in rectum and bladder V18.1 Gy with a median reduction of 2.2 % and 2.1 % respectively.

In our study, compromised NVB sparing showed the possibility for further NVB D_0.1cc_ reduction at the cost of dose to the PTV and prostate coverage. A similar PTV and prostate dose reduction is in line with recent studies in which dose de-escalation of (part of) the prostate in combination with a focal dose-escalating boost to the gross tumour volume is applied [25], [26]. Specifically, Teunissen et al. [18] demonstrated that using a lower PTV coverage aim compared to the PTV coverage used in this study (V_34.4Gy_ ≥ 80 % and V_34.4Gy_ ≥ 98 % respectively) facilitates NVB sparing to values below 32.8 Gy. For a subset of prostate cancer patients a further reduction in NVB D_0.1cc_ could be beneficial to reduce potential ED. A pareto front, as presented in this work, could help visualize the trade-off between PTV and prostate coverages and the accompanying levels of NVB sparing on patient-specific level. Combining an NTCP-model with a pareto front could further enhance the practical use of pareto fronts for shared decision making [27]. Planning automation allows to make this tool feasible in clinical practice.

Plans with a non-coplanar beam arrangement in general showed lower OAR doses (rectum, bladder V_18.1Gy_ and femurs) in non-NVBsparing, u-NVBsparing and especially c-NVBsparing settings, suggesting a benefit especially in challenging plans. Similar reductions in OAR doses have been shown in previous studies on coplanar versus non-coplanar beam arrangements for prostate radiotherapy. Bedford et al. [28] reported significantly lower rectum V_60Gy_ when using non-coplanar compared to coplanar beam arrangements for moderate hypofractionated radiotherapy. More recently, Rossi et al. [29] reported on statistically significant reductions of 5 % for rectum D_1cc_, 33 % for V_60Gy_, and 4 % for D_mean_ when using non-coplanar automated planning compared to VMAT automated planning for ultra-hypofractionated radiotherapy.

The Erasmus-iCycle optimizer generates fluence optimized plans which are therefore not deliverable. However, this does not impact the main findings of this study as previous studies have shown that these fluence optimized plans can be translated into clinically acceptable treatment plans without substantial loss of quality, using an in-house developed segmentation algorithm [30]. Even when accounting for scatter and transmission, segmentation is expected not to introduce systemic changes to the dose distributions for both beam arrangements [31].

A limitation of this work is the lack of certainty on which penile structures are involved in post-radiation ED. Studies on sparing penile bulb, corporal bodies and internal pudendal arteries have shown mixed results: Murray et al. reported a significant correlation between mean and maximum PB dose and ED [32], and Achard et al. reported on significantly more ED in patient receiving Crura Dmean > 4, 7 Gy and D2% >12 Gy [33]. In contrast, Zhang et al. [34] did not find any statistically significant correlation between dose in penile bulb and corporal bodies and ED. Spratt et al. [6] reported less ED after 2 years when sparing both internal pudendal arteries and proximal corporal bodies compared to validated model based predictions of ED rates after EBRT and radical prostatectomy. However, even with vessel sparing radiotherapy, patients expressed severe ED, which could suggest the importance of sparing other penile structures [7]. Studies on preserving erectile function after radiation therapy [8], [9] and prostatectomy [10], [35] suggest involvement of the NVB. Hence, this study is limited to sparing of bilateral NVB only. Important to note is that with the planning technique used in this study, the corporal bodies dose often remained far below the constraint used by Spratt et al. [6]. Furthermore, the planning technique used in our study, especially the non-coplanar beam arrangement, seems well suited to spare other OARs close to the target (e.g. internal pudendal arteries), as appears evident from the low doses in the OARs (mostly far lower than the constraints). Overall, although which penile structure best correlates to post-radiation ED remains unknown, the sparing of NVBs can be achieved with minimal costs and should therefore be considered.

This study focused solely on bilateral NVB sparing without investigating unilateral sparing. A previous study on MRI-guided adaptive radiotherapy showed that, in the presence of accurate intraprostatic lesion visibility, unilateral NVB sparing was more often feasible than bilateral sparing [36]. The authors found that for 102 men with no or minimal ED (IIEF-5 ≥ 17) at baseline, 68 % of patients could be spared unilaterally while only 20 % could be spared bilaterally. In a large meta-analyses on NVB sparing during prostatectomy, a clinical benefit for unilateral NVB sparing was found compared to non NVB sparing [37]. However bilateral NVB sparing was found to be most favourable for ED post-prostatectomy. The clinical impact of unilateral NVB sparing during radiotherapy is, however, yet unknown and could be subject of further research.

To conclude, in this study NVB sparing without compromising clinical constraints was demonstrated. Plans with a non-coplanar beam arrangement, although generally outperforming plans with coplanar beam arrangements, did not result in large clinical differences, except when looking OAR dose in medium dose ranges (i.e. rectum and bladder V_18.1Gy_). Additionally, these NVB dose reductions, although statistically significant, still need validation in prospective clinical trials to put their clinical relevance into perspective (e.g. POTEN-C trial, NCT03525262; ERECT trial, NCT04861194).

## Declaration of competing interest

The authors declare that they have no known competing financial interests or personal relationships that could have appeared to influence the work reported in this paper.

## Figures and Tables

**Table A1:** Full list of delineation definition. CTV: clinical target volume; PTV: planning target volume.

Organ	Delineation guideline
CTV	Entire prostate as visualized on the MRI, adjusted to planning CT-scan where needed
PTV	CTV + 3 mm margin
Rectum	Defined as a solid structure, including the lumen and rectal wall, extending from the anus to the rectosigmoid junction. The rectosigmoid junction is usually denoted as where the rectum turns forward and laterally.
Bladder	defined as a solid structure including the bladder wall and lumen
Urethra	the prostatic urethra is defined as the lumen/mucosal interface, extending from bladder neck to the membranous urethra
Femoral heads	Femoral heads are to be outlined from their most cranial aspect to the bottom of the curvature of the femoral head (i.e. exclude the femoral neck)
Bowel	Above rectum within 2 cm of PTV, may be outlined as a ‘bowel bag’
Scrotum	The bilateral testes should therefore be used as a ‘blocking structure’.
Neurovascular bundles	Separate structures within 1 cm of the PTV volume, as visualized on the MRI. Particular care will be taken to delineate these structures as they course into the genitourinary diaphragm caudally past the penile bulb until their termination in erectile tissue.

**Table B1:** Clinical planning protocol as used in the PACE-trial (5). PTV: planning target volume; Gy: Gray; CTV: clinical target volume.

**Target volume**	**Dose to target volume**

PTV	36.25 Gy ≥ 95 %
CTV	40 Gy ≥ 95 %

Organ at risk	Dose volume constraints	

	*Dose (Gy)*	*Maximum volume (% or cc)*

Rectum	18.1	50 %
	29	20 %
	36	2 cc
Bladder	18.1	40 %
	37	10 cc
Femoral heads	14.5	5 %
Bowel	18.1	5 cc
	30	1 cc
Urethra	42	50 %
Scrotum	Blocking structure (no beams passing through it are allowed)	

**Table B2:** Reference wish-list. PTV: planning target volume; Gy: Gray; gEUD: generalised Equivalent Uniform Dose; LTCP: Logarithmic Tumor Control Probability; ^a^ = PTV excluding Urethra; ^b^ = Maximum dose constraints were set lower than clinical protocol to account for voxel sampling in the optimization; ^c^ = Dose in 2 cm ring directly inside body contour; ^d^ = Prostate volume excluding Urethra; ^e^ dose in patient volume in between shells at 3 cm from the PTV and 2 cm inside the body contour.

Constraints				
	*Structure*	*Type*	*Limit*	*Parameters*
	PTV_opt_*^a^*	Maximum	47 Gy	
	Rectum	gEUD	28 Gy	α = 12
	Bowel	Maximum	30 Gy*^b^*	
	Urethra	gEUD	41.8 Gy	α = 2
	Bladder	gEUD	36.5 Gy	α = 8
	Scrotum	Maximum	1.5 Gy	
	Shell 3 mm from PTV	Maximum	36.25 Gy	
	Shell 3 cm from PTV	Maximum	18 Gy	
	Ring 2 cm from PTV	Maximum	23 Gy	
	Entrance dose*^c^*	maximum	18 GY	

Objectives				
*Priority*	*Structure*	*Type*	*Goal*	*Parameters*

1	PTV_opt_	LTCP	0.09	D_c_ = 35.25 Gy, α = 0.8, sufficient = 0.09
2	Prostate_opt_*^d^*	LTCP	0.18	D_c_ = 39.89 Gy, α = 0.9, sufficient = 0.18
3	Femur Left	Maximum	15 Gy	sufficient = 15 Gy
3	Femur Right	Maximum	15 Gy	sufficient = 15 Gy
4	Rectum	LTCP	0 Gy	D_c_ = 32 Gy, α = -0.3
5	Bladder	LTCP	0 Gy	D_c_ = 34 Gy, α = 0.1
6	Rectum	Mean	0 Gy	
7	Bladder	Mean	0 Gy	
8	Urethra	Mean	0 Gy	
9	Dose Bath*^e^*	Mean	0 Gy	

**Table C1:** Planned dose comparison on relevant dosimetric parameters between non NVB sparing and uncompromised NVB sparing plans for both coplanar and non-coplanar beam arrangements. Statistical significance between coplanar and non-coplanar plans was determined using Wilcoxon signed rank test. IQR = interquartile range; PTV = planning target volume; NVB = Neurovascular Bundles (left and right were combined); Gy = Gray;

			**Non NVB sparing plans**			**Uncompromised NVB sparing plans**		
**Structure**	**Parameter**		**Coplanar**	**Non-coplanar**		**Coplanar**	**Non-coplanar**	
	(PACE-constraint^23^)	Unit	Median (IQR)	Median (IQR)	P-value	Median (IQR)	Median (IQR)	P-value
**PTV**	**V_36.25Gy_** (>95 %)	%	97.3 (97.0–97.7)	97.2 (97.0–97.6)	<0.001	95.6 (95.2–95.8)	95.4 (95.1–95.7)	<0.001
**Prostate**	**V_40Gy_** (>95 %)	%	97.7 (97.4–98.2)	97.7 (97.4–98.1)	0.13	95.9 (95.8–96.2)	95.8 (95.8–96.2)	0.14
**NVB**	**D_0.1cc_**	Gy	42.6 (42.4–43.2)	43.3 (43.1–43.5)	<0.001	38.9 (38.3–39.1)	38.9 (38.3–39.1)	0.18
	**D_mean_**	Gy	30.0 (28.9–31.6)	30.2 (28.1–30.7)	0.048	25.6 (23.4–26.2)	24.7 (22.5–26.0)	<0.001
**Urethra**	**V_42Gy_** (<50 %)	%	40.1 (36.6–44.3)	41.8 (38.5–43.4)	0.11	40.8 (38.4–46.4)	41.6 (39.5–44.3)	1
**Rectum**	**V_36Gy_** (<2cc)	cc	0.23 (0.07–0.44)	0.20 (0.06–0.38)	<0.001	0.45 (0.24–0.66)	0.41 (0.23–0.65)	<0.001
	**V_29Gy_** (<20 %)	%	3.2 (2.4–5.1)	3.0 (2.3–4.8)	<0.001	3.9 (2.8–5.6)	3.6 (2.6–5.3)	<0.001
	**V_18.1Gy_** (<50 %)	%	10.3 (6.8–15.0)	9.4 (6.2–13.4)	<0.001	12.8 (8.3–16.3)	10.6 (7.1–14.3)	<0.001
**Bladder**	**V_37Gy_** (<10 cc)	cc	2.90 (1.91–3.78)	3.00 (1.92–3.98)	0.003	3.28 (2.45–4.41)	3.36 (2.45–4.51)	0.29
	**V_18.1Gy_** (<40 %)	%	13.7 (12.1–17.8)	11.6 (10.6–13.1)	<0.001	14.9 (12.8–19.9)	12.8 (11.6–14.6)	<0.001
**Bowel**	**V_30Gy_** (<1cc)	cc	0.0 (0.0–0.0)	0.0 (0.0–0.0)	1.0	0.0 (0.0–0.0)	0.0 (0.0–0.0)	1.0
	**V_18.1Gy_** (<5cc)	cc	0.0 (0.0–0.0)	0.0 (0.0–0.0)	1.0	0.0 (0.0–0.0)	0.0 (0.0–0.0)	1.0
**Femurs**	**V_14.5Gy_** (<5%)	%	2.3 (1.5–3.1)	0.2 (0.0–0.5)	<0.001	2.0 (1.2–3.2)	0.0 (0.0–0.2)	<0.001

**Table E1:** Coplanar planned dose comparison on relevant dosimetric parameters between the 7 compromised NVB sparing plans generated by stepwise lowering of neurovascular bundle constraint. PTV = planning target volume; NVB = neurovascular bundles (left and right were combined); Femurs: left and right were combined; Gy = Gray.

**Structure**	**Parameter**	**Dmax 50 Gy**	**Dmax** **40 Gy**	**Dmax** **37.5 Gy**	**Dmax** **35 Gy**	**Dmax** **32.5 Gy**	**Dmax 30 Gy**	**Dmax 25 Gy**
	(PACE/ *unit*)	*Median* *(IQR)*	*Median* *(IQR)*	*Median* *(IQR)*	*Median* *(IQR)*	*Median* *(IQR)*	*Median* *(IQR)*	*Median* *(IQR)*
**PTV**	**V_36.25Gy_** *(≥95 %)*	97.3 (97.0–97.7)	97.3 (97.0–97.7)	95.3 (94.9–95.9)	90.6 (89.0–91.9)	84.8 (82.2–87.0)	78.6 (75.2–80.9)	45.4 (39.9–50.9)
**Prostate**	**V_40Gy_** *(≥95 %)*	97.7 (97.5–98.2)	97.5 (97.1–97.7)	95.5 (95.1–95.8)	86.9 (85.4–90.3)	80.9 (78.2–84.7)	67.2 (59.4–70.3)	15.0 (7.01–19.1)
**NVB**	**D_0.1cc_** *(Gy)*	42.6 (42.5–43.2)	40.1 (40.0–40.1)	37.9 (37.7–37.9)	35.3 (35.3–35.4)	32.9 (32.8–32.9)	30.4 (30.3–30.4)	25.4 (25.3–25.4)
**Urethra**	**V_42Gy_** *(≤50 %)*	40.2 (36.8–44.4)	41.1 (37.4–44.4)	42.8 (39.7–46.2)	42.7 (39.2–46.5)	42.3 (40.3–44.7)	41.9 (39.0–45.9)	1.1 (0.0–6.2)
**Rectum**	**V_36Gy_** *(≤2cc)*	0.23 (0.08–0.46)	0.26 (0.10–0.51)	0.70 (0.32–0.77)	0.94 (0.61–1.41)	0.61 (0.31–1.08)	0.20 (0.06–0.52)	0.0 (0.0–0.0)
	**V_29Gy_** *(≤20 %)*	3.3 (2.3–5.1)	3.3 (2.3–5.2)	4.3 (2.7–6.5)	8.7 (6.6–10.0)	10.4 (7.2–11.9)	10.2 (6.4–12.0)	3.7 (2.7–4.7)
	**V_18.1Gy_** *(≤50 %)*	10.5 (6.7–15.0)	10.4 (6.7–14.8)	14.3 (9.0–19.2)	23.4 (19.1–27.4)	28.3 (22.2–32.9)	30.4 (23.3–35.9)	28.7 (21.1–32.9)
**Bladder**	**V_37Gy_** *(≤10 cc*)	2.90 (1.89–3.80)	2.92 (1.92–4.17)	3.87 (2.54–4.88)	4.65 (3.66–5.69)	4.00 (3.20–5.16)	3.00 (2.34–3.97)	0.51 (0.13–0.96)
	**V_18.1Gy_** *(≤40 %)*	13.7 (12.1–17.8)	14.0 (12.1–17.8)	15.7 (13.1–18.8)	18.0 (16.2–22.4)	18.6 (16.8–23.3)	18.5 (17.1–23.4)	17.2 (15.4–19.9)
**Bowel**	**V_30Gy_** *(≤1cc)*	0.0 (0.0–0.0)	0.0 (0.0–0.0)	0.0 (0.0–0.0)	0.0 (0.0–0.0)	0.0 (0.0–0.0)	0.0 (0.0–0.0)	0.0 (0.0–0.0)
	**V_18.1Gy_** *(≤5cc)*	0.0 (0.0–0.0)	0.0 (0.0–0.0)	0.0 (0.0–0.0)	0.0 (0.0–0.0)	0.0 (0.0–0.0)	0.0 (0.0–0.0)	0.0 (0.0–0.0)
**Femurs**	**V_14.5Gy_** *(≤5%)*	2.4 (1.5–3.2)	2.2 (1.5–3.1)	1.0 (0.4–1.7)	0.5 (0.3–0.8)	0.3 (0.1–0.7)	0.3 (0.1–0.6)	0.3 (0.2–0.6)
